# Lipoxin receptor agonist and inhibition of LTA_4_ hydrolase prevent tight junction disruption caused by *P*. *aeruginosa* filtrate in airway epithelial cells

**DOI:** 10.1371/journal.pone.0287183

**Published:** 2023-07-05

**Authors:** Kameljit K. Kalsi, Sonya Jackson, Deborah L. Baines

**Affiliations:** 1 Institute for Infection and Immunity, St George’s University of London, London, United Kingdom; 2 Translational Science and Experimental Medicine Research and Early Development, Respiratory, Inflammation & Autoimmune (RIA), Biopharmaceuticals R&D, AstraZeneca, Gothenburg, Sweden; Universita Politecnica delle Marche, ITALY

## Abstract

Airway diseases can disrupt tight junction proteins, compromising the epithelial barrier and making it more permeable to pathogens. In people with pulmonary disease who are prone to infection with *Pseudomonas aeruginosa*, pro-inflammatory leukotrienes are increased and anti-inflammatory lipoxins are decreased. Upregulation of lipoxins is effective in counteracting inflammation and infection. However, whether combining a lipoxin receptor agonist with a specific leukotriene A_4_ hydrolase (LTA_4_H) inhibitor could enhance these protective effects has not to our knowledge been investigated. Therefore, we explored the effect of lipoxin receptor agonist BML-111 and JNJ26993135 a specific LTA_4_H inhibitor that prevents the production of pro-inflammatory LTB_4_ on tight junction proteins disrupted by *P*. *aeruginosa* filtrate (PAF) in human airway epithelial cell lines H441 and 16HBE-14o. Pre-treatment with BML-111 prevented an increase in epithelial permeability induced by PAF and conserved ZO-1 and claudin-1 at the cell junctions. JNJ26993135 similarly prevented the increased permeability induced by PAF, restored ZO-1 and E-cadherin and reduced IL-8 but not IL-6. Cells pre-treated with BML-111 plus JNJ26993135 restored TEER and permeability, ZO-1 and claudin-1 to the cell junctions. Taken together, these data indicate that the combination of a lipoxin receptor agonist with a LTA_4_H inhibitor could provide a more potent therapy.

## Introduction

Infections with *Pseudomonas aeruginosa* are an ongoing challenge to the clinical community. *P*. *aeruginosa* is an opportunistic pathogen of the airway, urinary tract and skin. In the airway it can cause chronic infections in people with compromised host defence mechanism for example in patients with pneumonia in long term care [1] or in patients with pre-existing diseases such as in cystic fibrosis (CF) [2]. It has been indicated that *P*. *aeruginosa* resides in the lower airway of more than 50% of adult CF patients (UK Cystic Fibrosis Registry Annual Data Report 2020, cysticfibrosis.org.uk/registryreports) [3].

The surface of the airways are lined with epithelial cells which form a barrier between the lumen and the interstitium. The cells of the epithelium are linked by junctional proteins. Anchoring the cell-to-cell adhesion are the adherens junctions which include E-cadherin and the catenin family of proteins [4]. Tight junctions are protein complexes that are formed between adjacent epithelial cells and are localised to the apical/luminal domain. The complexes consist of transmembrane proteins that include scaffolding proteins such as zonula occludens (ZO-1), claudins, occludin and junctional adhesion molecules. Together they regulate paracellular movement of ions and molecules between the apical/luminal and basolateral/interstitial domains of the epithelium [5]. Disruption of the tight junctions leads to changes in the permeability of the epithelium. This can result in a loss of epithelial barrier function and contribute to disease pathology.

During infection, the toxins associated with the virulence of *P*. *aeruginosa* includes exotoxin A, exoenzyme S, pyocyanins and elastase [6] have been shown to increase epithelial permeability by disrupting tight junction proteins [7]. A study using real time 3-dimensional microscopy found that bacteria can transmigrate the epithelial barrier at specific points where the tight junctions are temporarily disrupted at sites of cell division or cell death. Penetration of epithelial barrier by the bacteria can then lead to systemic infection [5]. The enhanced permeability to proteins, decreased wound closure and cleavage of Junctional Adhesion Molecule-A (JAM-A) has been attributed to the virulence of ExoA. These are released by *P*. *aeruginosa* through the actions of disintegrin and metalloproteinase 17 (ADAM17) and are responsible for the release of ectodomains [8]. *P*. *aeruginosa* also activates toll-like receptors (TLR2 and TLR4) triggering further inflammatory response pathways that affect epithelial permeability [9].

The defence mechanisms triggered by the host in response to bacterial infection can result in the release of many mediators both pro-inflammatory and anti-inflammatory. The arachidonic acid pathway, a precursor to prostaglandins, is central to the formation of mediators such as pro-inflammatory leukotrienes B_4_ (LTB_4_) and the anti-inflammatory lipoxins. It has been found that in the lungs of patients with chronic obstructive pulmonary disease (COPD), asthma [10] and cystic fibrosis disease [11, 12] levels of lipoxin are reduced and LTB_4_ increased [10]. Treatment of normal and CF airway epithelial cells with lipoxin A_4_ (LXA_4_) suppressed the disruption of tight junctions caused by infection with *P*. *aeruginosa* and attenuated bacterial colonization [13]. The use of stable LXA_4_ analogs and receptor agonists have shown promising results for the treatment of inflammatory diseases [14, 15] and inhaled BML-111, a lipoxin receptor agonist, has been tested in children with acute asthma [16]. Reducing the formation of LTB_4_ could also provide another method for reducing the inflammatory response. Several inhibitors have been formulated to interact with this pathway, including antagonists of LTB_4_ receptors (BLT1 and BLT2) and inhibitors of LTA_4_ hydrolase, which is a rate limiting enzyme in the generation of LTB_4_. The off-target effects of BLT1 and BLT2 on proliferator activator receptors (PPARs) has limited the clinical use of these compounds [17]. LTA_4_ hydrolase converts LTA_4_ to LTB_4_ by epoxide hydrolase activity [18]. Initial results with specific inhibitors targeting LTA_4_ hydrolase (JNJ40929837) found that LTB_4_ formation was inhibited but pro-inflammatory LTC_4_ production was increased. More promising was the LTA_4_ hydrolase inhibitor JNJ26993135 which was found to decrease airway inflammation in rodent models and not produce pro-inflammatory bi-products [19].

We hypothesised that BML-111, a LXA_4_ receptor agonist, and JNJ26993135 independently, or together, will prevent *P*. *aeruginosa* induced disruption of tight junctions and increased epithelial permeability. We used human airway H441 adenocarcinoma cells grown at air-liquid interface and 16HBE-14o immortalised with origin-of-replication defective SV40 plasmid (pSVori-) cell line grown as submerged cultures to achieve high resistance monolayers. All cell models form polarised airway epithelial monolayers that express functional tight junctions that modulate the barrier function.

## Materials and methods

### Epithelial cell culture

Human adenocarcinoma airway epithelial cells H441 from American Type Culture Collection (ATCC) were cultured in RPMI-1640 media with 10% foetal calf serum (FCS) (Invitrogen, UK), 10 mM glucose, 2 mM glutamate, 1 mM sodium pyruvate, 10 μg/mL insulin, 5 μg/mL transferrin, 7 ng/mL sodium selenite, 100 U/mL penicillin and 100 μg/mL streptomycin [20]. 16HBE14o- bronchial epithelial cell line was kindly obtained from Dr Bettina Schock, Queens University, Belfast and were cultured in Minimum Essential Medium (MEM) supplemented with 10% FCS. Cells were grown on flasks and transwells coated with 10 μg/ml human plasma fibronectin (Millipore, UK), 100 μg/ml bovine serum albumin (Sigma, UK) and 50 μg/ml Type 1 bovine collagen (Sigma, UK). H441 cells were seeded at 100,000 cells per transwell onto clear Transwell® (Costar) inserts (1.12 cm^2^ area, 0.45-μm pore size) and grown at air-liquid-interface (ALI) to form confluent polarised monolayers as described [21]. H441 cells were studied 10–14 days post seeding. 16HBE14o- were seeded at 200,000 cells per Transwell and grown submerged for 9 days to form polarised monolayers. Transepithelial electrical resistance TEER was measured using an electrovoltometer (EVOM) with chopstick electrodes (WPI, UK) using the manufacturers protocol. In brief, the electrodes were placed either side of the cell layer. A current was passed and the voltage measured. From this, resistance was calculated in Ω.cm^2^. The last three readings were taken from each well and the average calculated.

TEER was corrected for the resistance of blank inserts and the culture medium. Monolayers were pre-treated bilaterally with lipoxin agonist (BML-111, 50 nmol/L or 200 nmol/L) or leukotriene A_4_ hydrolase inhibitor JNJ 26993135 (30–300 nmol/L) in serum free media 30 min prior to the addition of *P*. *aeruginosa* filtrate as previously described [22, 23]. In latter experiments the combined effect of BML-111 and JNJ 26993135 were conducted on H441 monolayers.

### Pseudomonas aeruginosa filtrate

The laboratory strain *P*. *aeruginosa* PA01 was used to treat the apical/luminal surface of airway epithelial cells. A single colony of PA01 was incubated overnight in RPMI to form a confluent culture of approximately 5x10^9^ colony forming units (CFU)/ml. *P*. *aeruginosa* filtrate (PAF) was prepared by boiling for 10 minutes, centrifuging (5500 x *g* for 30 minutes) and then filtering through a 0.45 μm pore filter [24]. PAF (100 μl) was applied to the apical surface of polarised epithelial cells for 24h.

### Permeability assay

After TEER measurements, culture medium was replaced with Hanks Balanced Salt Solution (HBSS) (Sigma-Aldrich, UK) and incubated for 30 minutes, the apical solution was replaced with 0.5 mL HBSS with 10 μmol/L Na-fluorescein (MW = 367 Da, Sigma-Aldrich) which we used to indicate the epithelial permeability to small solutes as described by Ren, H *et al*. 2016 [25]. Samples (100 μl) were removed from the basolateral bath at time intervals of 0, 30, 60 and 90 minutes. Fluorescence was measured in black, 96-well plates using a GloMax fluorescence plate reader with excitation and emission wavelengths of 460 nm and 515 nm respectively. The calculation for measuring the permeability coefficients used the following equation: P_app_ = ((dC/dt)V/(AC_0_)), where dC/dt is the slope of the linear fit of the concentration of fluorophore /time, V is the volume of HBSS in the basolateral chamber, A is the surface area of the membrane (1.12 cm^2^), and C_0_ is the concentration of fluorescein added to the apical compartment (10 μmol/L) [25].

### Immunofluorescence microscopy

Tight junctions were examined by immunofluorescence staining. Cells were fixed with methanol:acetone for 10 min at room temperature and washed with PBS. Cells were blocked and permeabilized with 1% bovine serum albumin containing 0.1% Triton in Tris-buffered saline. Primary antibodies were as follows: mouse anti ZO-1, clone ZO1-1A12 (Thermofisher, UK); rabbit anti occludin polyclonal antibody (Santa Cruz Biotechnology); mouse anti-claudin-1, clone 2H10D10 (Thermofisher, UK); and mouse anti-E-cadherin, clone 36/E-cadherin (BD Biosciences, UK). All antibodies were diluted to 1:100 in blocking buffer. Cells were incubated with primary antibodies at room temperature for 1 hour, washed with PBS then secondary antibody was added (anti-mouse Alexa Fluor 594 or anti-rabbit Alexa Fluor 488 at 1:100 dilution) for 30 min at room temperature. After further washing with PBS, nuclei were stained with 4’,6-diamino-2-phenylindole (DAPI) (1:200; Sigma, UK) for 10 min. Transwell membranes were then cut out with a clean scalpel and mounted onto slides with Fluorsave mounting medium (Millipore, UK). Confocal microscopy was carried out by using a Nikon A1R microscope under oil immersion at 63x. Images were acquired and analysed using Nikon NIS-Elements C software. For stack images sequential images were acquired with ~0.5 μm steps. To aid comparisons an equal number of horizontal slices with the same vertical depth from apical to basolateral were acquired under identical exposure parameters. Images were exported to Fiji ImageJ for analysis. Pixel intensity was calculated using a macro from ImageJ where multiple vertical parallel lines were created on an image, each line is 50 pixels apart, in order to measure the pixel density along each line (S1 Fig).

### Cytokines

Cell free basal media was collected and stored at -80°C for cytokine analysis from epithelial cells treated with PAF and JNJ 26993135. The concentrations of IL-6 and IL-8 were measured using duoset sandwich ELISA kits (DY206 and DY208, R&D Systems, Abingdon, UK). Cell supernatants were diluted in the appropriate reagent diluent and kits were used following the manufacturer’s instructions. Concentrations of IL-6 and IL-8 were calculated by plotting the best fit curve of the standard absorbance vs the standard concentration, the concentration in the sample was corrected for any dilution. The assay range for IL-6 is 9.4–600 pg/mL and for IL-8 is 31.2–2,000 pg/ml.

### Statistical analysis

For statistical analysis, *P* ≤ 0.05 was taken as significant. Where normal distribution could be assessed data were analysed by ANOVA with post hoc Tukey’s test where indicated. Other data were subject to non-parametric testing. Data are shown as mean ± SD, where *n*  =  the number of independent experiments.

## Results

### BML-111 attenuates the effects of PAF on TEER and permeability of H441 polarised epithelial monolayers

**Fig 1:**
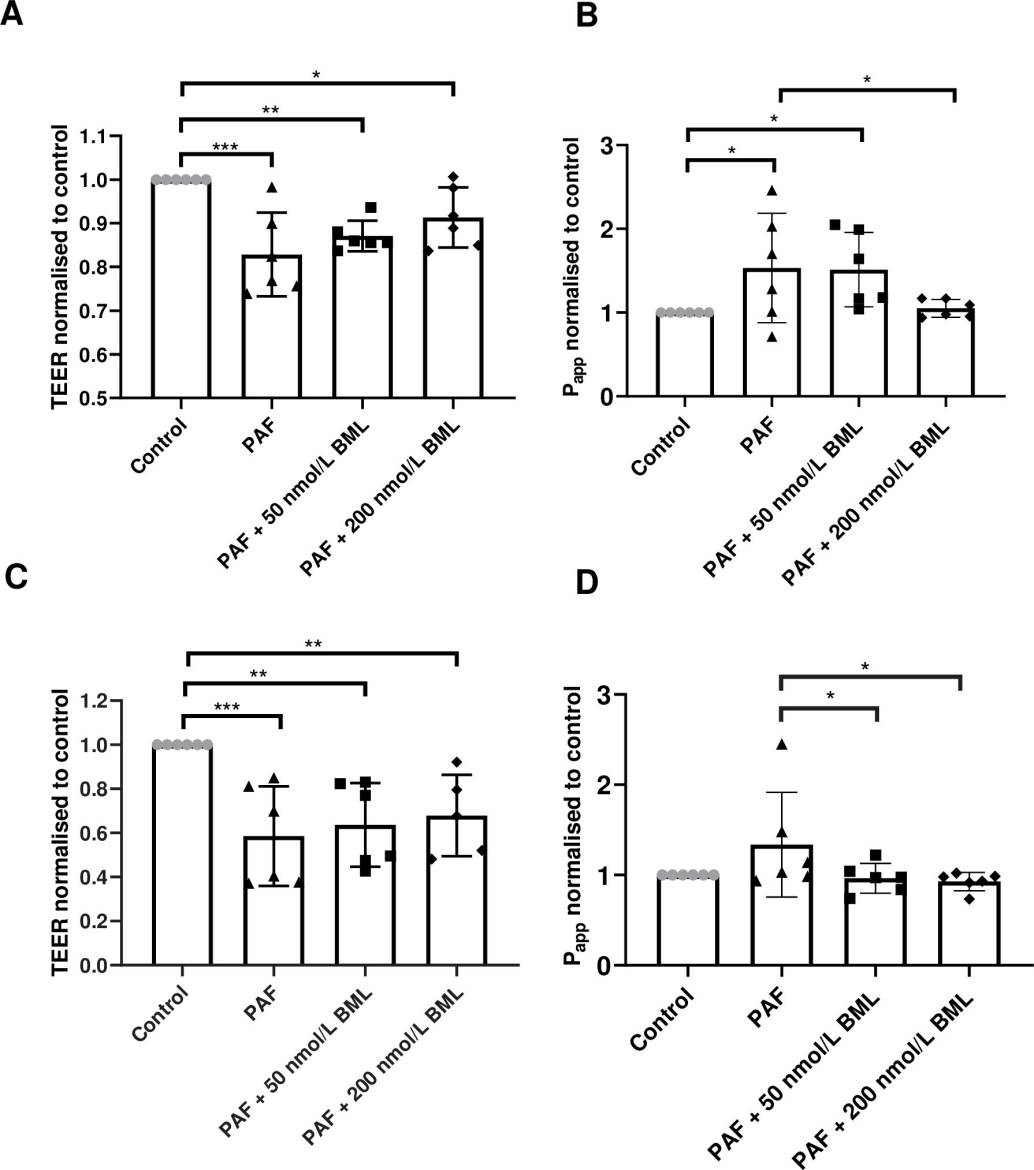
Effect of BML-111 on transepithelial resistance (TEER) and permeability of H441 cells and 16HBE-14o. *(A*) TEER of H441 polarised monolayers before (control) or after treatment with *P*. *aeruginosa* filtrate (PAF) for 24h or PAF + 50 nmol/L BML-111 or PAF + 200 nmol/L BML-111. *(B)* Apical to basolateral permeability of H441 monolayers before (control) or after treatment with *P*. *aeruginosa* filtrate (PAF) for 24h or PAF + 50 nmol/L BML-111 or PAF + 200 nmol/L BML-111. *(C*) TEER of 16HBE14o- polarised monolayers before (control) or after treatment with *P*. *aeruginosa* filtrate (PAF) for 24h or PAF + 50 nmol/L BML-111 or PAF + 200 nmol/L BML-111. *(D)* Apical to basolateral permeability of 16HBE14o- polarised monolayers before (control) or after treatment with *P*. *aeruginosa* filtrate (PAF) for 24h or PAF + 50 nmol/L BML-111 or PAF + 200 nmol/L BML-111. Data are shown normalised to control, means ± SD (bars) with individual data points. Statistical analysis was conducted using one-way ANOVA followed by Tukey’s multiple comparison test. Significant differences as shown *P<0.05, **P<0.01, ***P<0.001 all n = 6.

The addition of 100 μl of undiluted PAF to the apical surface of polarised H441 cells produced a significant decrease in TEER from 166 ± 10 to 128 ± 6 Ω.cm^2^ after 24 hrs or to 0.83 ± 0.09 of control values (*P* < 0.0001; n = 4–6; Fig 1A). TEER after pre-treatment with 50 nmol/L BML-111 was 0.87 ± 0.04 of control and 200 nmol/L BML-111 attenuated the reduction of TEER to 0.91 ± 0.07 of control but both remained lower than control TEER values (*P* < 0.01; n = 4–6 and *P* < 0.05; n = 6 respectively). Preliminary data with 200 nmol/L of BML-111 alone did not show differences compared to control.

To compare the changes in TEER induced after PAF exposure with changes to epithelial permeability and barrier function, the permeability coefficient (P_app_) was measured using the passage of Na-fluorescein, from the apical to the basolateral compartment over 90 mins. There was a significant increase in P_app_ after the addition of PAF from 3.9 ± 0.9 x 10^−7^ cm/s to 8.9 ± 5 x 10^−7^ cm/s, or (1.53 ± 0.65, of control values, *P* < 0.05; n = 6; Fig 1B). The increase of P_app_ was attenuated by pre-treatment with 200 nmol/L BML-111 to 1.05 ± 0.11 of control (*P* < 0.05; n = 6) but not with 50 nmol/L BML-111 (which remained at 1.51 ± 0.44 of control, n = 6).

### BML-111 does not prevent the decrease in TEER but attenuates the increase in P_app_ induced by PAF in 16HBE14o- polarised epithelial monolayers

Treatment of 16HBE14o- cells grown as submerged polarised monolayers, with PAF significantly decreased TEER from 393 ± 60 to 206 ± 46 Ω.cm^2^ after 24 hrs, or 0.59 ± 0.22 of control values (*P* < 0.0001; n = 5–6; Fig 1C). Pre-treatment with 50 nmol/L or 200 nmol/L BML-111 did not attenuate the decrease in TEER which remained at 0.64 ± 0.19 and 0.68 ± 0.18 of control values respectively (*P* < 0.0001; n = 5–6).

Permeability was significantly increased with PAF treatment from 3.7 ± 0.6 x 10^−7^ cm/s to 5.4 ± 1.6 x 10^−7^ cm/s, or 1.34 ± 0.58 of control values, (*P* < 0.05; n = 5; Fig 1D). Both 50 nmol/L and 200 nmol/L BML-111 significantly decreased P_app_ to 0.96 ± 0.16 and 0.93 ± 0.10 of control respectively (*P* < 0.05 and *P* < 0.01; n = 5–6, compared to PAF).

### BML-111 prevents the PAF-induced reduction of ZO-1 and occludin at the cell junctions in H441 polarised monolayers

**Fig 2:**
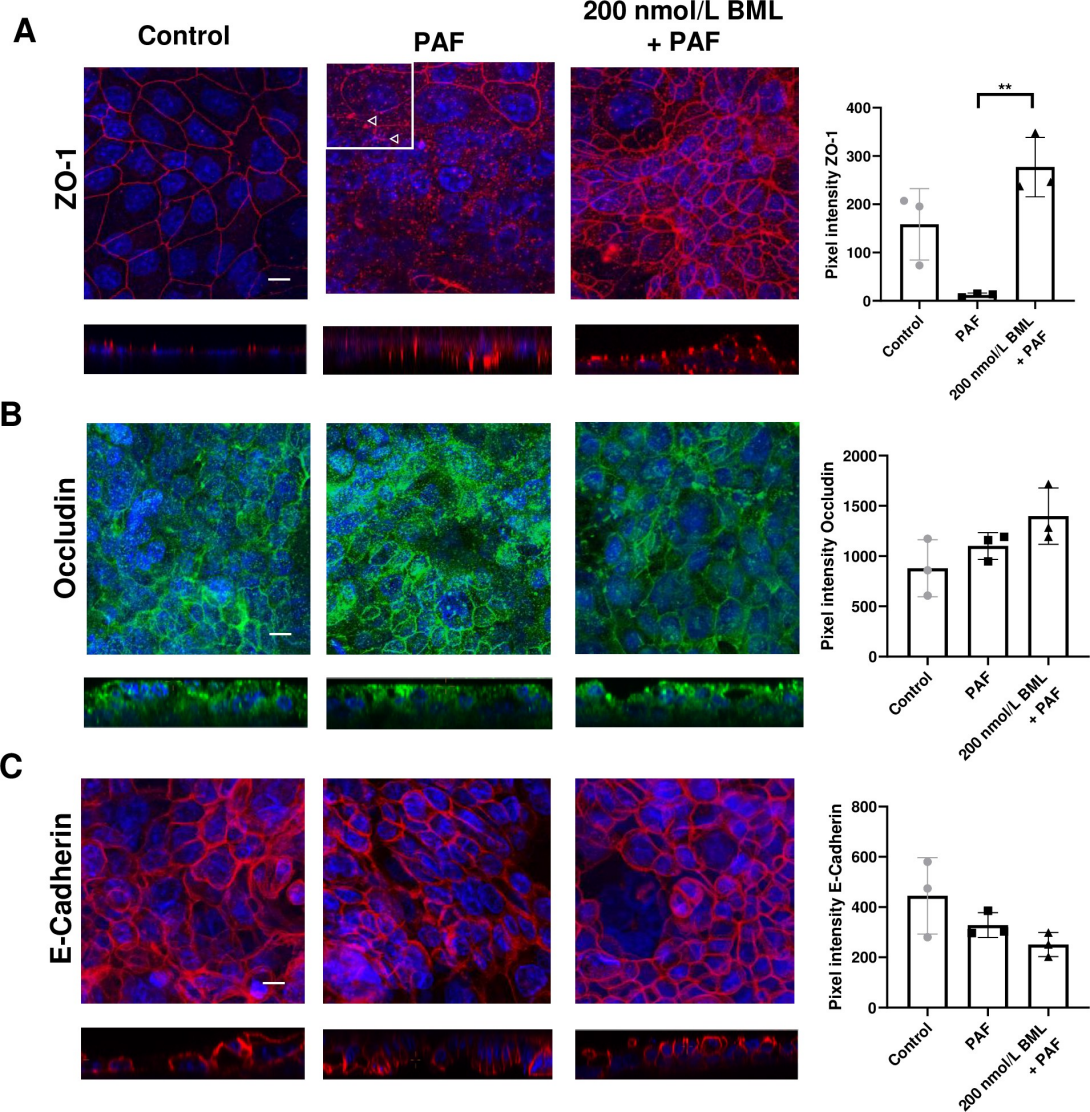
ZO-1 localisation at the tight junctions is disrupted by PAF and restored by BML-111. Composite X/Y confocal images of H441 polarised monolayers untreated (control) or treated with *P*. *aeruginosa* filtrate (PAF) for 24h or PAF + 200 nmol/L BML-111 (BML) immunostained for *(A)* ZO-1 (red). The white highlighted area shows regions (arrows) where ZO-1 immunostaining at the tight junction was not contiguous. *(B)* Occludin (green) and *(C)* E-cadherin (red). Nuclei were stained with Dapi (blue). All images were obtained at ×63 magnification. X/Z cross-sectional images are shown beneath with the apical surface oriented to the top. Scale bars (20 μm) are shown to the bottom left of the control image only but apply to all images. Graphical data of pixel intensities at the tight junctions for each protein are shown to the right of the images as means ± SD (bars) with individual data points. Non-parametric statistical analysis was carried out using nonparametric Kruskal‐Wallis multiple comparison test. Significant differences as shown **P<0.01, all n = 3.

Immunofluorescence staining of proteins associated with tight junctions in H441 epithelial monolayers (Fig 2A and S2 Fig) indicated that PAF altered ZO-1 localisation at the apical cellular junctions with only small patches of contiguous fluorescent staining remaining. Pixel intensity plots revealed a decrease in ZO-1 fluorescence at the cell junctions in cells treated with PAF to 12 ± 4 compared to 158 ± 74 pixels in control. Treatment with 200 nmol/L BML-111 + PAF restored ZO-1 fluorescence to 277 ± 61 pixels (*P* < 0.01; n = 3) compared to PAF. Occludin fluorescence at the cell junctions was observed to be partially disrupted with PAF whereas there was little effect on E-cadherin localisation (Fig 2B & 2C) and pixel intensity at the cell junctions was unchanged.

### BML-111 prevents PAF-induced reduction in ZO-1, Claudin-1 and enhances E-cadherin at the cell junctions in 16HBE-14o polarised monolayers

**Fig 3:**
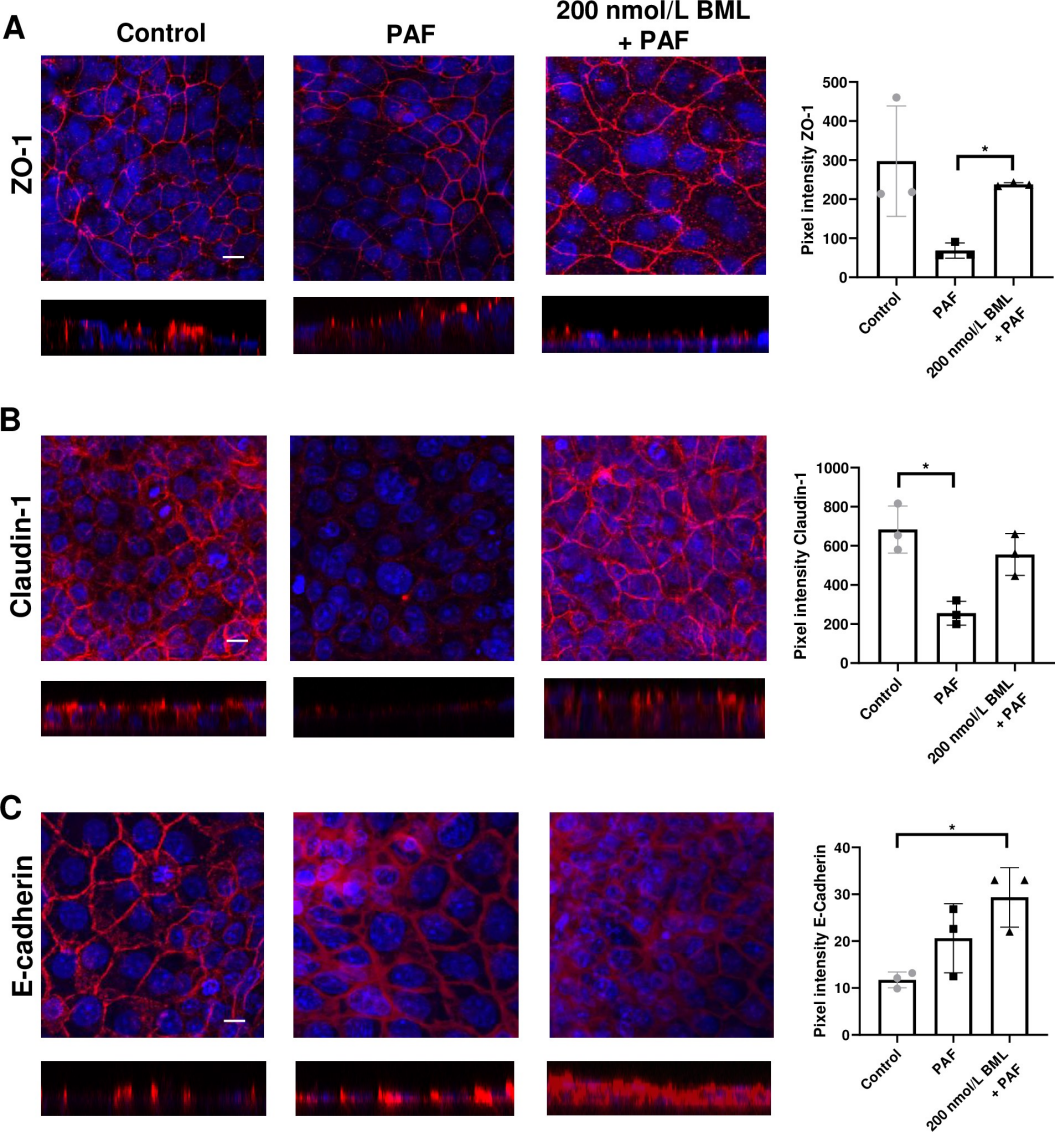
Claudin-1 localisation at the tight junctions is disrupted by PAF and restored by BML-111. Composite X/Y confocal images of 16HBE14o- polarised monolayers untreated (control) or treated with *P*. *aeruginosa* filtrate (PAF) for 24h or PAF + 200 nmol/L BML-111 (BML) immunostained for *(A)* ZO-1 (red), *(B)* Claudin-1 (red), *(C)* E-cadherin (red). Nuclei were stained with Dapi (blue). All images were obtained at ×63 magnification. X/Z cross-sectional images are shown beneath with the apical surface oriented to the top. Scale bars (20 μm) are shown to the bottom left of the control image only but apply to all images. Graphical data of pixel intensities at the tight junctions for each protein are shown to the right of the images as means ± SD (bars) with individual data points. Non-parametric statistical analysis was carried out using nonparametric Kruskal‐Wallis multiple comparison test. Significant differences as shown, *P<0.05, all n = 3.

To investigate the effects of PAF in 16HBE14o- epithelial monolayers, cells were immunostained for ZO-1, claudin-1 and E-cadherin. Unfortunately, we could not obtain good staining of 16HBE14o- using the occludin antiserum. Therefore, we studied claudin-1. These cells form monolayers of higher resistance than H441 cells. We observed that both ZO-1 and claudin-1 fluorescence at the cell junctions was reduced after PAF treatment (Fig 3A, 3B and S3 Fig) compared to control. For ZO-1, pixel intensity was 297 ± 141 in control, reduced to 68 ± 20 after exposure to PAF, and was restored to 237 ± 5 after pretreatment with BML-111 (P<0.05, n = 3). For claudin-1, pixel intensity at the cell junctions was 683 ± 120, reduced to 255 ± 61 after treatment with PAF (*P* < 0.05; n = 3) and was restored to 555 ± 107 pixels after pre-treatment with BML-111. Interestingly, observed immunofluorescence of E-cadherin (Fig 3C) appeared to be increased but pixel intensity was unchanged, 12 ± 2 in control and 21 ± 7 pixels after treatment with PAF. There was an increase in E-cadherin fluorescence in the cells after pre-treatment with BML-111 and pixel intensity was 29 ± 6 (*P* < 0.05; n = 3) compared to control but x/z images indicated that the protein was throughout the cell and not specifically localised to the cell junctions.

### JNJ26993135 does not prevent the decrease in TEER but attenuates the increase in P_app_ induced by PAF in H441 polarised epithelial monolayers

**Fig 4:**
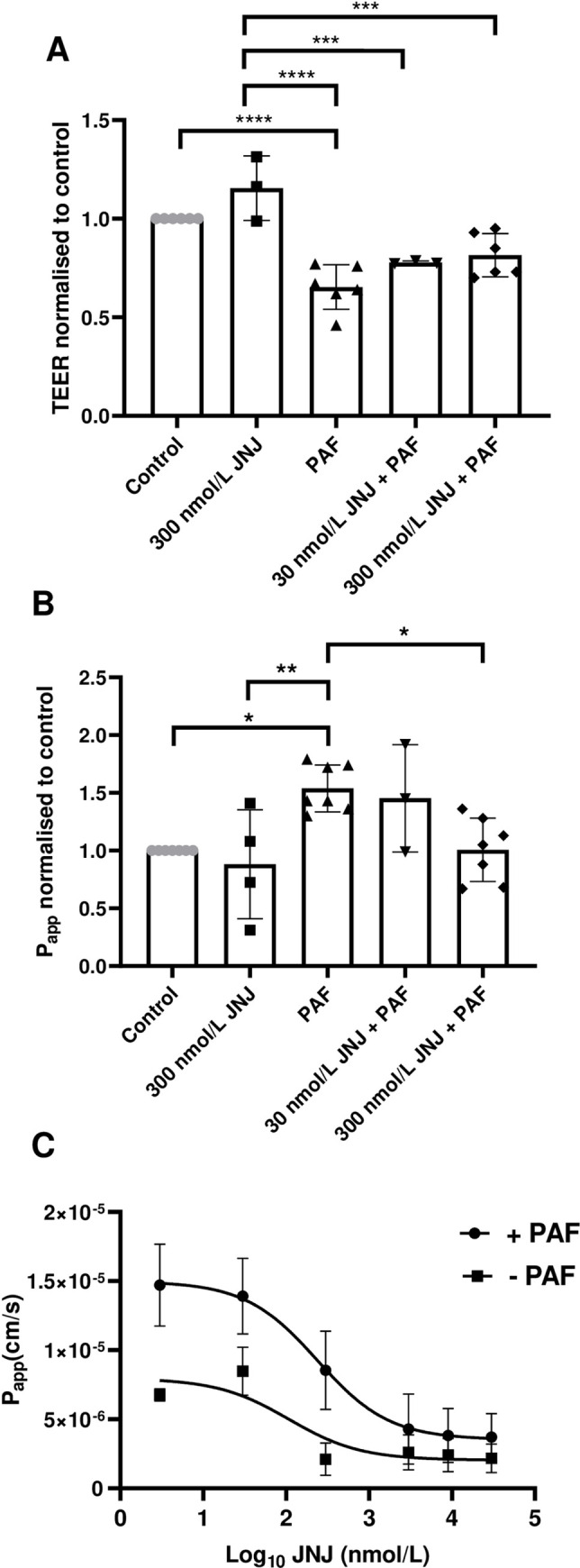
JNJ26993135 prevents PAF induced increase in permeability and improves TEER in H441 monolayers. (*A*) TEER of H441 polarised monolayers before (control) or after treatment with *P*. *aeruginosa* filtrate (PAF) for 24h or 300 nmol/L JNJ26993135 (JNJ) or PAF + 30 nmol/L JNJ or PAF + 300 nmol/L JNJ. *(B)* Apical to basolateral permeability of H441 monolayers before (control) or after treatment with *P*. *aeruginosa* filtrate (PAF) for 24h or 300 nmol/L JNJ or PAF + 30 nmol/L JNJ or PAF + 300 nmol/L JNJ. *(C)* Dose response curves for JNJ are shown on permeability across H441 monolayers in the presence or absence of PAF. Data are shown normalised to control, means ± SD (bars) with individual data points. Statistical analysis was conducted using one-way ANOVA followed by Tukey’s multiple comparison test. Significant differences as shown *P<0.05, **P<0.01, ***P<0.001, ****P<0.0001, n = 3–6. Data for the dose response of JNJ are mean ± SEM (n = 3–6).

To assess whether JNJ26993135 pre-treatment could prevent the decrease in TEER or the increase in permeability induced by PAF, H441 polarised monolayers were treated with 300 nmol/L JNJ26993135 alone or 30 nmol/L or 300 nmol/L JNJ26993135 thirty minutes prior to PAF exposure. TEER was reduced in cells exposed to PAF compared to control or 300 nmol/L JNJ26993135 alone (*P* < 0.0001, n = 3–6) (Fig 4A). Inhibition of LTA_4_ hydrolase with JNJ26993135 did not prevent the reduction in TEER caused by PAF. However, the increase in P_app_ induced by PAF was mitigated by 300 nmol/L JNJ26993135 (*P* < 0.05, n = 6) but not 30 nmol/L JNJ26993135 (Fig 4B). A dose response curve of JNJ26993135 in the presence or absence of PAF (Fig 4C) on permeability was more robust after treatment with PAF, but the LogIC_50_ of JNJ for the different treatments was not different.

### JNJ26993135 prevents the PAF-induced loss of ZO-1 and E-cadherin at the cell junctions

**Fig 5:**
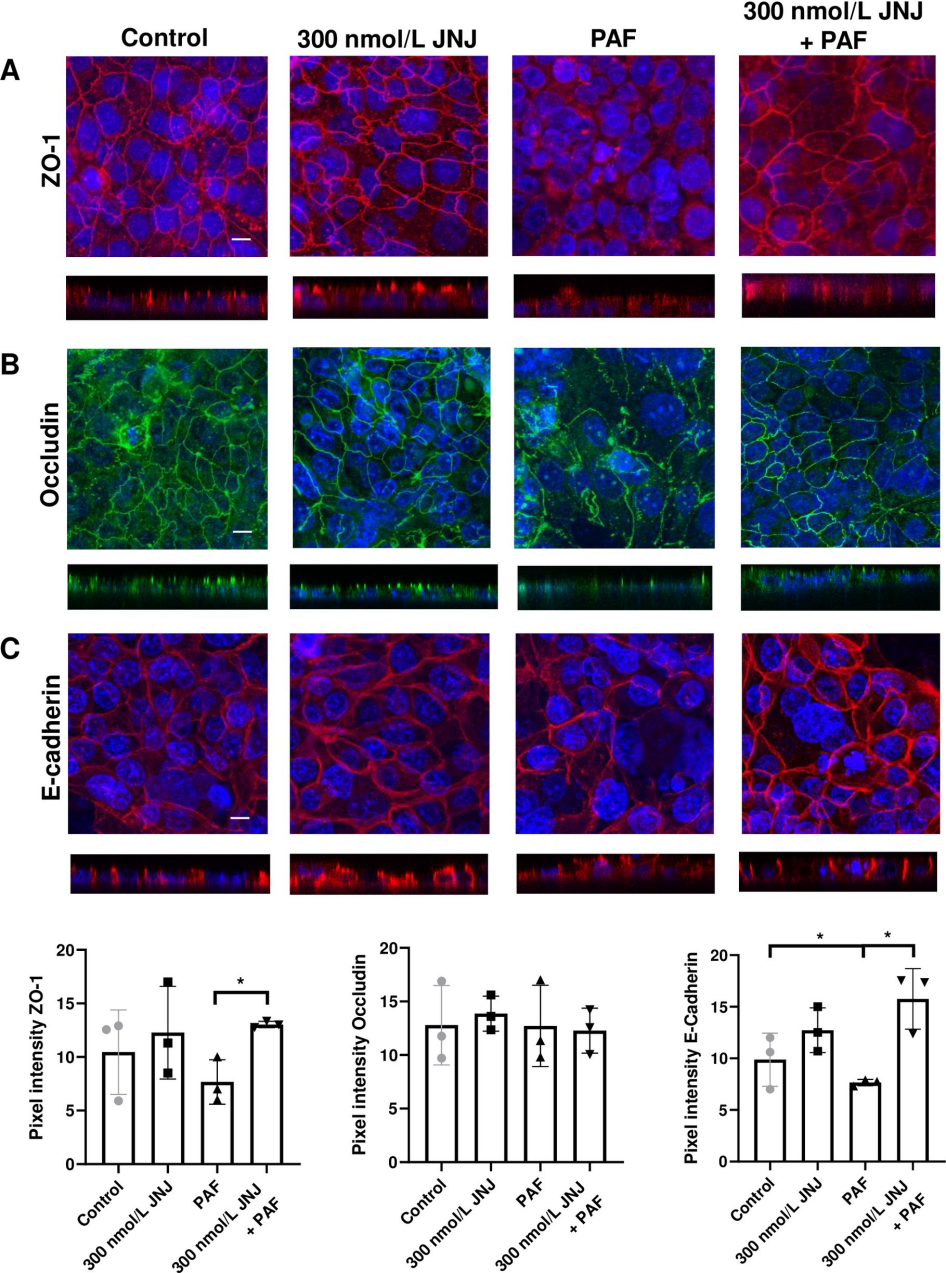
JNJ26993135 enhances ZO-1 and Occludin but only partially protects tight junctions disrupted by PAF. Composite X/Y confocal images of H441 polarised monolayers untreated (control) or treated with *P*. *aeruginosa* filtrate (PAF) for 24h or 300 nmol/L JNJ26993135 (JNJ) or PAF + 300 nmol/L JNJ immunostained for *(A)* ZO-1 (red), *(B)* Occludin (green), *(C)* E-cadherin (red). Nuclei were stained with Dapi (blue). All images were obtained at ×63 magnification. X/Z cross-sectional images are shown beneath with the apical surface oriented to the top. Scale bars (20 μm) are shown to the bottom right of the control image only but apply to all images. Graphical data of pixel intensities at the tight junctions for each protein are shown at the bottom of the images as means ± SD (bars) with individual data points. Non-parametric statistical analysis was carried out using nonparametric Kruskal‐Wallis multiple comparison test. Significant differences as shown, *P<0.05, all n = 3.

Incubating H441 polarised monolayers with 300 nmol/L JNJ26993135 enhanced the observed ZO-1 and occludin fluorescence at the apical cell junctions compared to control cells (Fig 5A, 5B and S4 Fig) but pixel intensity plots showed that these were unchanged. ZO-1 was significantly increased from 7 ± 2 pixels with PAF to 13 ± 0.2 pixels (*P* < 0.05; n = 3) in cells pre-treated with 300 nmol/L JNJ26993135 prior to PAF exposure. The lateral fluorescence for E-cadherin was also increased with JNJ26993135 (Fig 5C). Pixel intensity plot for E-cadherin showed a significant decrease with PAF to 7 ± 0.3 pixels from 9 ± 2 pixels compared to control (*P* < 0.05; n = 3). Pre-treatment with 300 nmol/L JNJ26993135 prior to PAF exposure augmented pixel intensity to 16 ± 3 pixels compared to PAF (*P* < 0.05; n = 3). The loss in fluorescent signal for ZO-1 and E-cadherin at the cellular junctions induced by PAF was prevented by 300 nmol/L JNJ26993135.

### JNJ26993135 prevents the PAF-induced release of IL-8 but not IL-6

**Fig 6:**
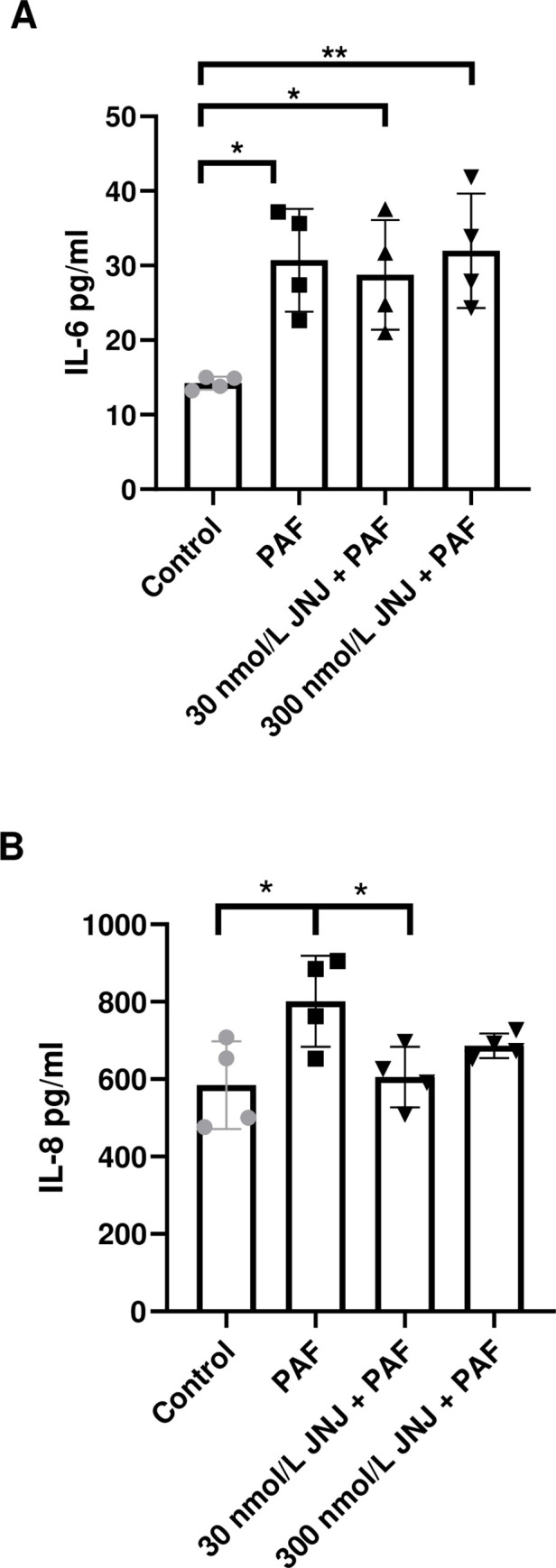
PAF stimulates the release of IL-6 and IL-8 from H441 cells. (*A)* IL-6 and *(B*) IL-8 present in the basolateral medium from H441 polarised monolayers before (control) or after treatment with *P*. *aeruginosa* filtrate (PAF) for 24h or PAF + 30 nmol/L JNJ 26993135 (JNJ) or PAF + 300 nmol/L JNJ. IL-6 and IL-8 were measured in basolateral media from polarised H441 cells after treatments. Data are shown as mean± SD (bars) with individual data points. Statistical analysis was conducted using one-way ANOVA followed by Tukey’s multiple comparison test. Significant differences as shown *P<0.05, **P<0.01, all n = 4.

There was a significant induction of IL-6 and IL-8 release into the cell medium of H441 polarised cell monolayers after 24h treatment with PAF (*P* < 0.05; n = 4; Fig 6A & 6B). Pre-treatment with either 30 nmol/L or 300 nmol/L JNJ26993135 did not prevent the increase in IL-6 release stimulated by PAF. In contrast 30 nmol/L JNJ26993135 prevented the increase in IL-8 release induced by PAF (*P* < 0.05; n = 4; Fig 6B).

### Combined effects of lipoxin agonist and LTA_4_ hydrolase inhibitor

**Fig 7:**
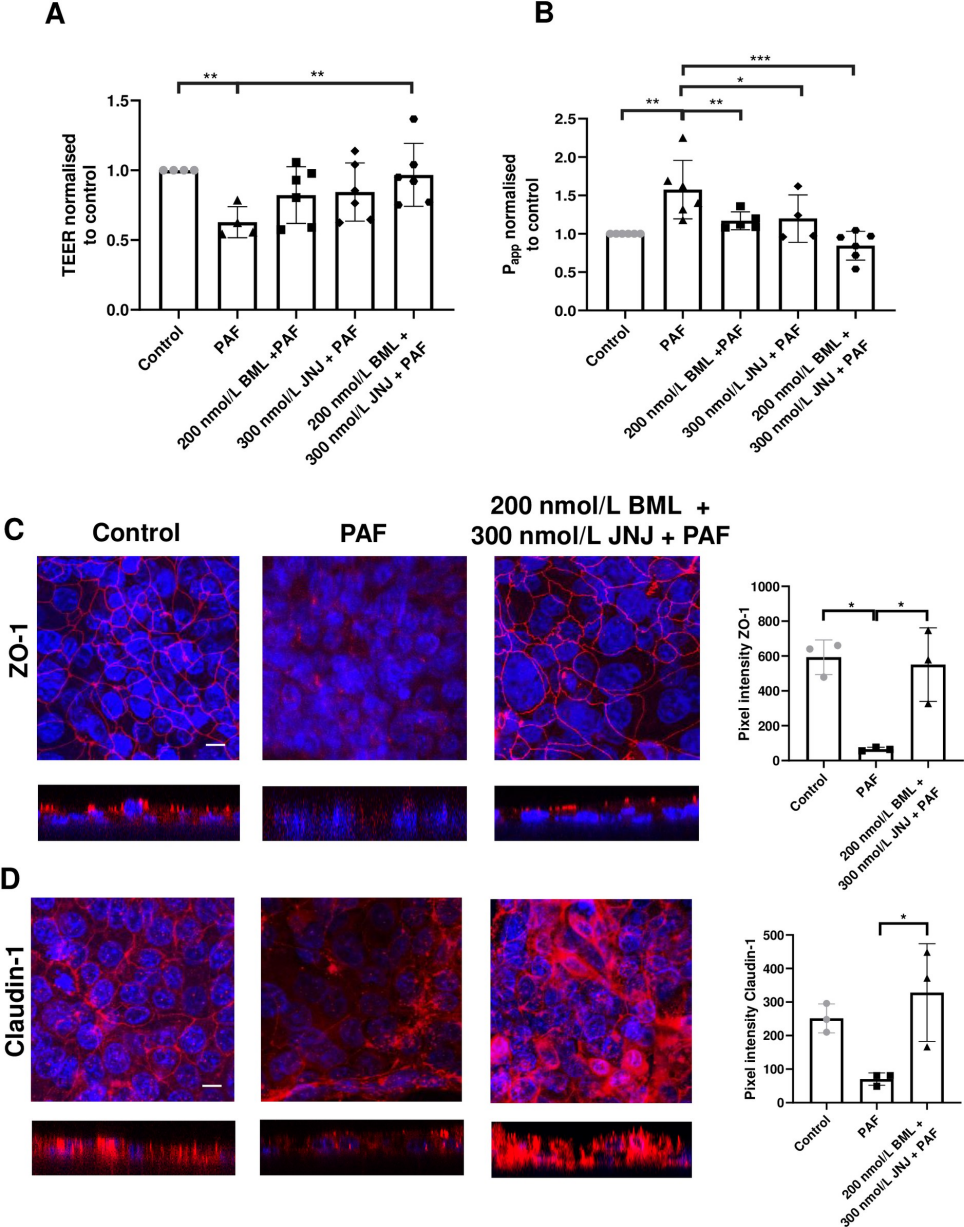
JNJ26993135 + BML-111 decreases permeability and enhances ZO-1 and claudin-1 induced by PAF but does not change TEER. *(A*) TEER and *(B)* apical to basolateral permeability of H441 polarised monolayers before (control) or after treatment with *P*. *aeruginosa* filtrate (PAF) for 24h or PAF + 200 nmol/L BML-111 (BML) or PAF + 300 nmol/L JNJ 26993135 (JNJ) or PAF + 200 nmol/L BML-111 (BML) + 300 nmol/L JNJ. Data are shown normalised to control, means ± SD (bars) with individual data points. Statistical analysis was conducted using one-way ANOVA followed by Tukey’s multiple comparison test. Significant differences as shown **P < 0.01, ***P < 0.001, n = 4–6. Composite X/Y confocal images of H441 polarised monolayers untreated (control) or treated with *P*. *aeruginosa* filtrate (PAF) for 24h or PAF + 200 nmol/L BML-111 (BML) + 300 nmol/L JNJ immunostained for *(C)* ZO-1 (red) and *(D)* Claudin 1 (red). Nuclei were stained with Dapi (blue). All images were obtained at ×63 magnification. X/Z cross-sectional images are shown beneath with the apical surface oriented to the top. Scale bars (20 μm) are shown to the bottom left of the control image only but apply to all images. Graphical data of pixel intensities at the tight junctions for each protein are shown to the right of the images as means ± SD (bars) with individual data points. Non-parametric statistical analysis was carried out using nonparametric Kruskal‐Wallis multiple comparison test. Significant differences as shown *P < 0.05, all n = 3.

Since we showed that the lipoxin agonist BML-111 had a protective effect on maintaining the tight junctions and decreasing permeability induced by PAF, we questioned whether combination with the specific inhibitor of LTA_4_ hydrolase would enhance this protection further. We tested this by pre-treating H441 polarised monolayers with 200 nmol/L BML-111 plus 300 nmol/L JNJ26993135. PAF reduced TEER to 0.63 ± 0.11 of control values (*P < 0.05; n = 4; Fig 7A). Pre-treatment with BML-111 + JNJ26993135 prior to the addition of PAF prevented any reduction in TEER caused by PAF (0.95 ± 0.25, *P < 0.05; n = 5, compared to PAF). Permeability, normalised to control was significantly increased by PAF to 1.57 ± 0.38 (*P* < 0.01; n = 6; Fig 7B) which was prevented by pre-treatment with BML-111 and JNJ26993135 (0.84 ± 0.18, ***P < 0.001; n = 6, compared to PAF).

To assess the effects on the localisation of ZO-1 and claudin-1 (Fig 7C, 7D and S5 Fig), pixel intensity was quantified across images acquired from H441 polarised monolayers. The plots revealed a significant increase in pixel intensity of ZO-1 and claudin-1 at the cell junctions in monolayers pre-treated with BML-111 + JNJ26993135 prior to exposure to PAF than when exposed to PAF alone (*P* < 0.05; n = 3) respectively.

## Discussion

Infection with *P*. *aeruginosa* in the airway leads to inflammation particularly in tissue that is already injured or slow to repair. This compromises the barrier function of the airway epithelium [26]. Here we used two different human airway cell lines exposed to *P*. *aeruginosa* filtrate to test whether a lipoxin A_4_ receptor agonist BML-111 or LTA_4_ hydrolase inhibitor JNJ26993135 could restore barrier function [26].

We used H441 lung adenocarcinoma cells and 16HBE14o- bronchiolar epithelial cells. Culture conditions can affect TEER and in these experiments the cell lines provided models of relatively low and high TEER respectively. We used a filtrate of *P*. *aeruginosa* (PAF) [24] to standardise the effects and help reduce variability associated with co-culture with live bacteria. However, we continued to observe variability in the response to PAF between experiments. Exposure to PAF reduced TEER and increased epithelial permeability in both cell lines we studied. This was associated with reduced ZO-1 and claudin-1 localisation at the tight junctions consistent with previous observations [27]. Elastase and exotoxin A are the major virulence factors released by *P*. *aeruginosa* that are likely to be present in PAF [28]. Other known virulence factors are phospholipase C, protease A, exotoxins and cytotoxins, flagella and pili, pigment production, and QS regulatory system proteins are involved in transcriptional changes and formation of biofilm [29]. Elastases are known to down-regulate expression of occludin, claudins 1, 4 and 7 (but not E-cadherin which is located more towards the basal cellular domain compared to ZO-1 and occludin) Elastase can also disable PAR-2 (protease activated receptors-2) and induce PKC signalling which can also modify tight junction assembly and localisation by phosphorylation [28, 30].

In this study we found that the lipoxin A_4_ receptor agonist BML-111 did not restore the PAF induced reduction in TEER but did decrease permeability to Na-fluorescein. In both H441 and 16HBE14o- polarised monolayers these changes were associated with the preserved expression and localisation of ZO-1 at the tight junctions [31]. In 16HBE14o-, claudin-1 was also restored to the tight junction, whereas in H441 cells occludin remained disrupted. These findings are consistent with those described for lipoxin A_4_ which restored ZO-1 and claudin-1 expression in cystic fibrosis airway epithelial cells after *P*. *aeruginosa* exposure, supporting BML-111 action as a lipoxin A_4_ receptor agonist [13]. ZO-1 is critical for the co-ordination and assembly of tight junctions [32]. Claudin-1 has been shown to regulate permeability of molecules across airway epithelial cells [33], the epidermis and biliary ducts [34]. Thus, the restoration of these proteins to the tight junction would support a decrease in permeability to small solutes as we have shown. LXA_4_ has also been shown to modify airway ion transport through an inhibition of amiloride sensitive Na^+^ channel and stimulation of Cl^-^ currents [35].

Thus, the lack of effect of BML-111 on TEER may reflect changes to ion transport. Since transepithelial electrical resistance is a measure of resistance to the movement of ions across the epithelium including the paracellular pathway (diffusion) and transcellular (ion transport). It is possible that effects on pathways regulating small solute diffusion were not reflected in TEER because of the smaller size of ions compared to solutes and/or that the transcellular pathways for ion movement are unaffected. In our measurements of TEER we did not discriminate between paracellular and transcellular (which can be estimated through measurement of transepithelial potential difference).

A further aim of this study was to assess the effects on airway epithelial cell barrier function, of blocking leukotriene A_4_ hydrolase (LTA_4_ hydrolase), which is the rate limiting step for formation of LTB_4_. We used JNJ26993135 which specifically blocks the epoxide hydrolase activity of LTA_4_ while leaving the aminopeptidase activity of this bifunctional enzyme still active and able to degrade Pro-Gly-Pro (PGP). This is important in the lung as PGP is a neutrophil chemoattractant and is accumulated in CF, COPD and bronchiolitis obliterans syndrome [36]. JNJ26993135 was also shown to increase LXA_4_ levels 1–2 hours post administration [23] (S6 Fig). We found that 300 nmol/L of JNJ26993135 alone had no effect on TEER or permeability of H441 airway epithelial cells. However, in the presence of PAF while there was no effect on TEER, there was a reduction of small solute permeability with 300 nmol/L JNJ26993135. This concentration is towards the maximal reported for inhibition of LTB_4._ The IC_50_ for JNJ26993135 was 10 nmol/L in a mouse model of arachidonic acid induced ear inflammation and 339 nmol/L in blood to inhibit the production of LTB_4_ [23, 37]. Similar to BML-111, 300 nmol/L JNJ26993135 restored ZO-1 fluorescence the tight junction after exposure to PAF indicating a similar mechanism of action. Interestingly E-cadherin fluorescence at the tight junction was also enhanced with JNJ26993135 [37].

Incubation with PAF promoted the release of IL-6 and IL-8 from the airway epithelial cells. PAF may contain flaggelin (a structural protein of Pseudomonas flagella) which has been shown to induce release of IL-6 and IL-8 in bronchial airway (BEAS-2B) cells [23]. Blocking LTA_4_ hydrolase with JNJ26993135 attenuated IL-8 release but not IL-6. We have no further data to explain this finding. However, flaggelin was shown to promote the phosphorylation of p38 mitogen activated protein kinase (MAPK), extracellular signal-regulated kinase (ERK) and c-Jun N-terminal kinase (JNK). Both IL-6 and IL-8 were inhibited with a p38 inhibitor but only ERK inhibition reduced IL-8 [23]. Thus, it is possible that JNJ26993135 may differentially affect these signalling pathways. Lastly, in models of peritonitis, the effect of JNJ26993135 on the production of LXA_4_ was transient, lasting approximately 3 hours [22]. We hypothesised that inhibiting the formation of LTB_4,_ with JNJ26993135, together with the exogenous LXA_4_ receptor agonist BML-111 may better protect the barrier function after assault with PAF. The effect of BML-111 + JNJ26993135 on TEER and permeability was not additive but permeability was more consistently/robustly reduced. Similarly, the restoration of ZO-1 and claudin 1 at the tight junctions was more clearly demarked.

Taken together these data indicate that LXA_4_ receptor agonists and modifiers of the LTA_4_ hydrolase /LTB_4_ pathway can singularly or together mitigate the effect of *P*. *aeruginosa* filtrate on airway barrier function by reducing IL-8 release, permeability to small solutes and restoring ZO-1 and claudin-1 proteins to the tight junctions. There are limitations in using cell lines to investigate differences in tight junctions following treatments. More work is now required to delineate the action of JNJ26993135 on LXA_4,_ LTB_4_ and the signalling pathways involved particularly in primary cells, derived from cohorts with airway disease where epithelial barrier function or *Pseudomonas aeruginosa* plays a role in disease pathogenesis (e.g. asthma, cystic fibrosis). Nevertheless, we suggest that inhibition of LTB_4_ together with LXA_4_ receptor agonists may promote an augmentation of pro-resolving lipoxins and thus could be important future targets for further investigations.

## Supporting information

S1 Fig Macro for measuring tight junctions from Fiji ImageJ. (PPTX)

S2 Fig BML-111 restores PAF-reduced ZO-1 in H441 cells. (PPTX)

S3 Fig BML-111 restores PAF-reduced ZO-1 in 16HBE14o-. (PPTX)

S4 Fig JNJ26993135 restores PAF-reduced ZO-1 and E-cadherin at the tight junctions. (PPTX)

S5 Fig JNJ26993135 + BML-111 restores PAF-reduced ZO-1 and claudin-1 at the tight junctions. (PPTX)

S6 Fig Schematic of the proposed signalling mechanism of lipoxin and LTA_4_. (PPTX)
