# Supplementary material for: Lipoxin receptor agonist and inhibition of LTA4 hydrolase prevent tight junction disruption caused by P. aeruginosa filtrate in airway epithelial cells
Source: PLoS One. 2023 Jul 5;18(7):e0287183. doi: 10.1371/journal.pone.0287183 (PMC10321624; doi:10.1371/journal.pone.0287183)
Supplement: S1 Fig — (PPTX) [file pone.0287183.s001.pptx]

## Slide 1
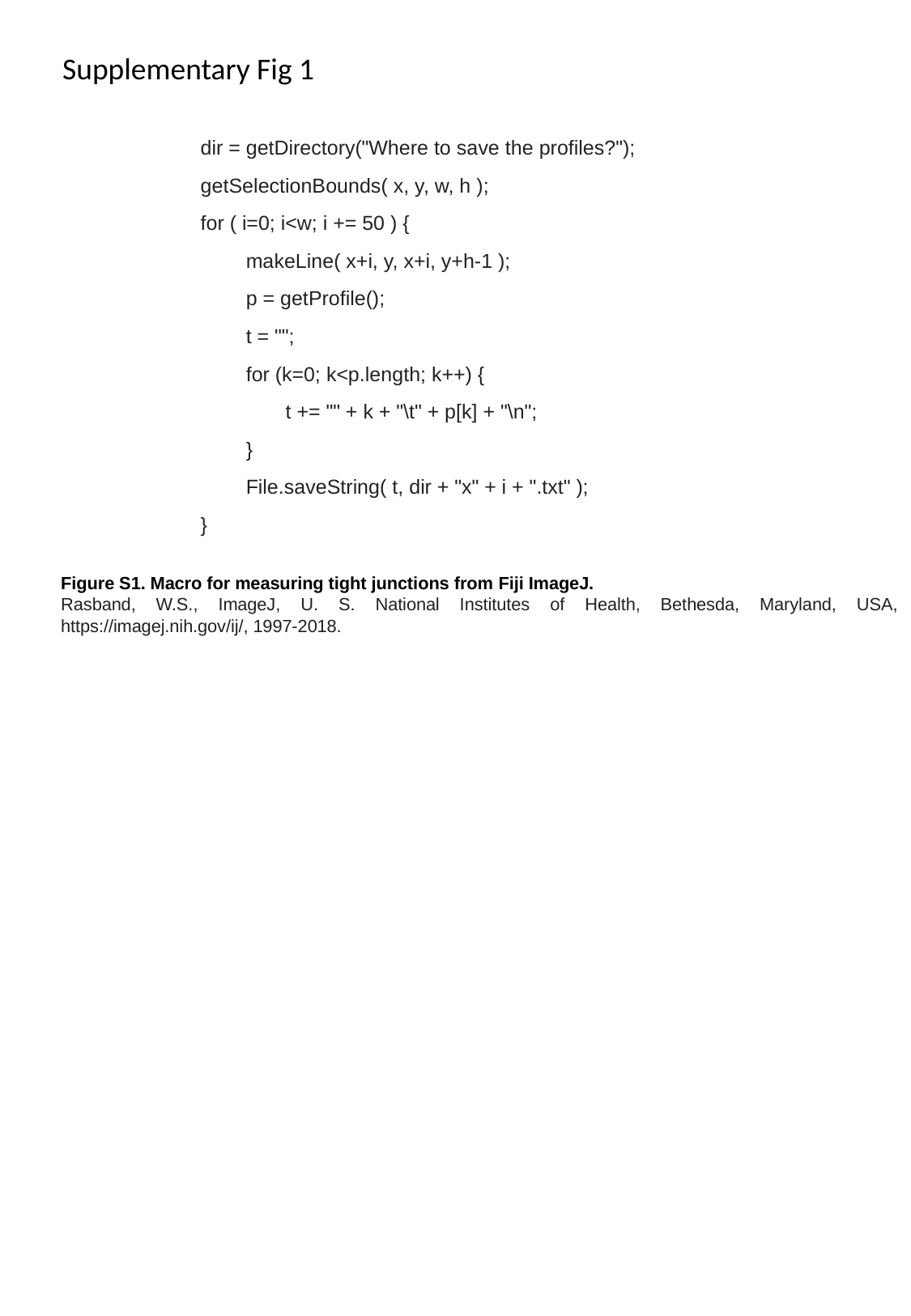

Supplementary Fig 1
dir = getDirectory("Where to save the profiles?");
getSelectionBounds( x, y, w, h );
for ( i=0; i<w; i += 50 ) {
        makeLine( x+i, y, x+i, y+h-1 );
        p = getProfile();
        t = "";
        for (k=0; k<p.length; k++) {
               t += "" + k + "\t" + p[k] + "\n";
        }
        File.saveString( t, dir + "x" + i + ".txt" );
}
Figure S1. Macro for measuring tight junctions from Fiji ImageJ.
Rasband, W.S., ImageJ, U. S. National Institutes of Health, Bethesda, Maryland, USA, https://imagej.nih.gov/ij/, 1997-2018.
