# Supplementary material for: Lipoxin receptor agonist and inhibition of LTA4 hydrolase prevent tight junction disruption caused by P. aeruginosa filtrate in airway epithelial cells
Source: PLoS One. 2023 Jul 5;18(7):e0287183. doi: 10.1371/journal.pone.0287183 (PMC10321624; doi:10.1371/journal.pone.0287183)
Supplement: S4 Fig — (PPTX) [file pone.0287183.s004.pptx]

## Slide 1
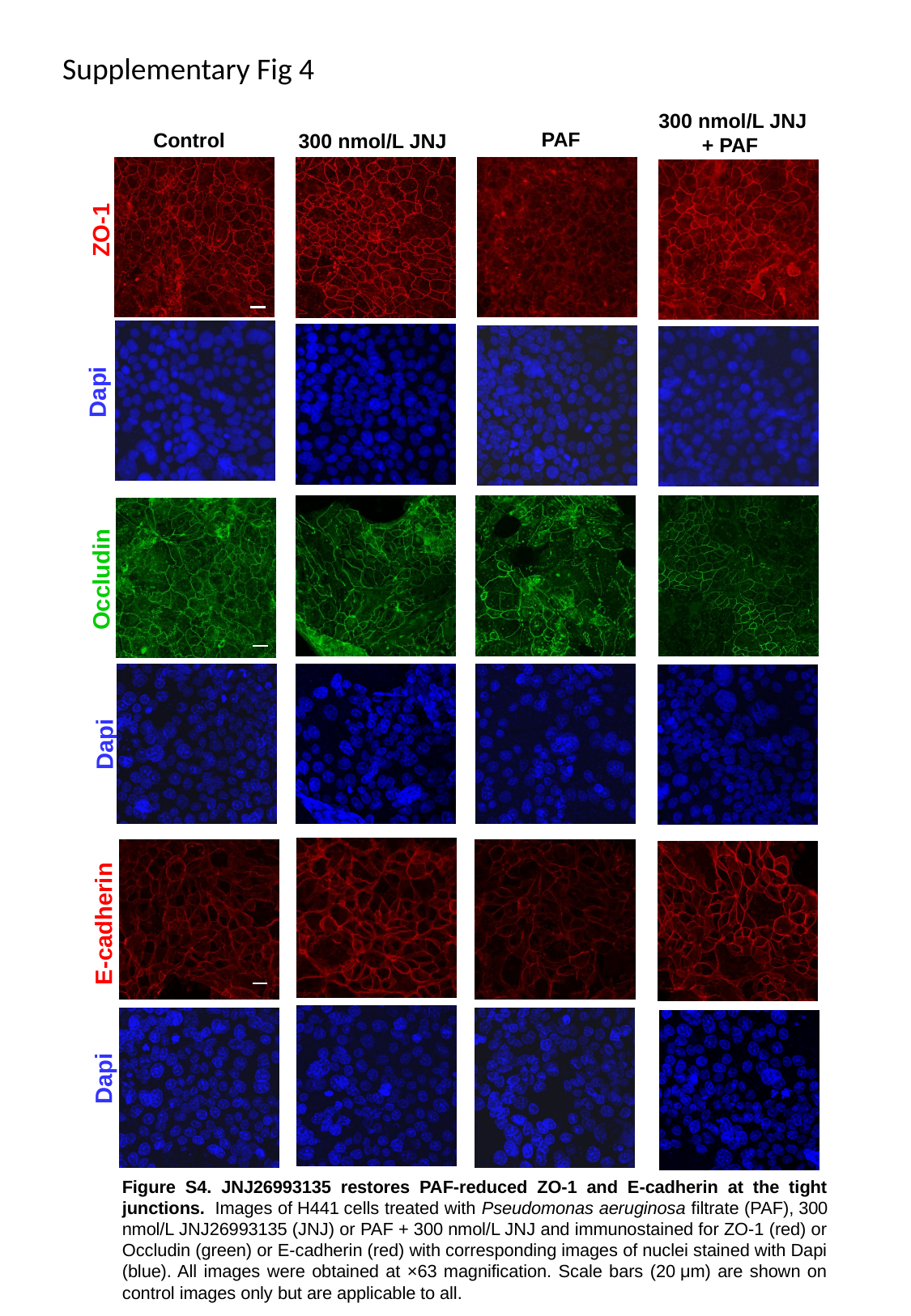

Supplementary Fig 4
300 nmol/L JNJ + PAF
PAF
Control
300 nmol/L JNJ
ZO-1
Dapi
Occludin
Dapi
E-cadherin
Dapi
Figure S4. JNJ26993135 restores PAF-reduced ZO-1 and E-cadherin at the tight junctions.  Images of H441 cells treated with Pseudomonas aeruginosa filtrate (PAF), 300 nmol/L JNJ26993135 (JNJ) or PAF + 300 nmol/L JNJ and immunostained for ZO-1 (red) or Occludin (green) or E-cadherin (red) with corresponding images of nuclei stained with Dapi (blue). All images were obtained at ×63 magnification. Scale bars (20 μm) are shown on control images only but are applicable to all.
