# Supplementary material for: Lipoxin receptor agonist and inhibition of LTA4 hydrolase prevent tight junction disruption caused by P. aeruginosa filtrate in airway epithelial cells
Source: PLoS One. 2023 Jul 5;18(7):e0287183. doi: 10.1371/journal.pone.0287183 (PMC10321624; doi:10.1371/journal.pone.0287183)
Supplement: S5 Fig — (PPTX) [file pone.0287183.s005.pptx]

## Slide 1
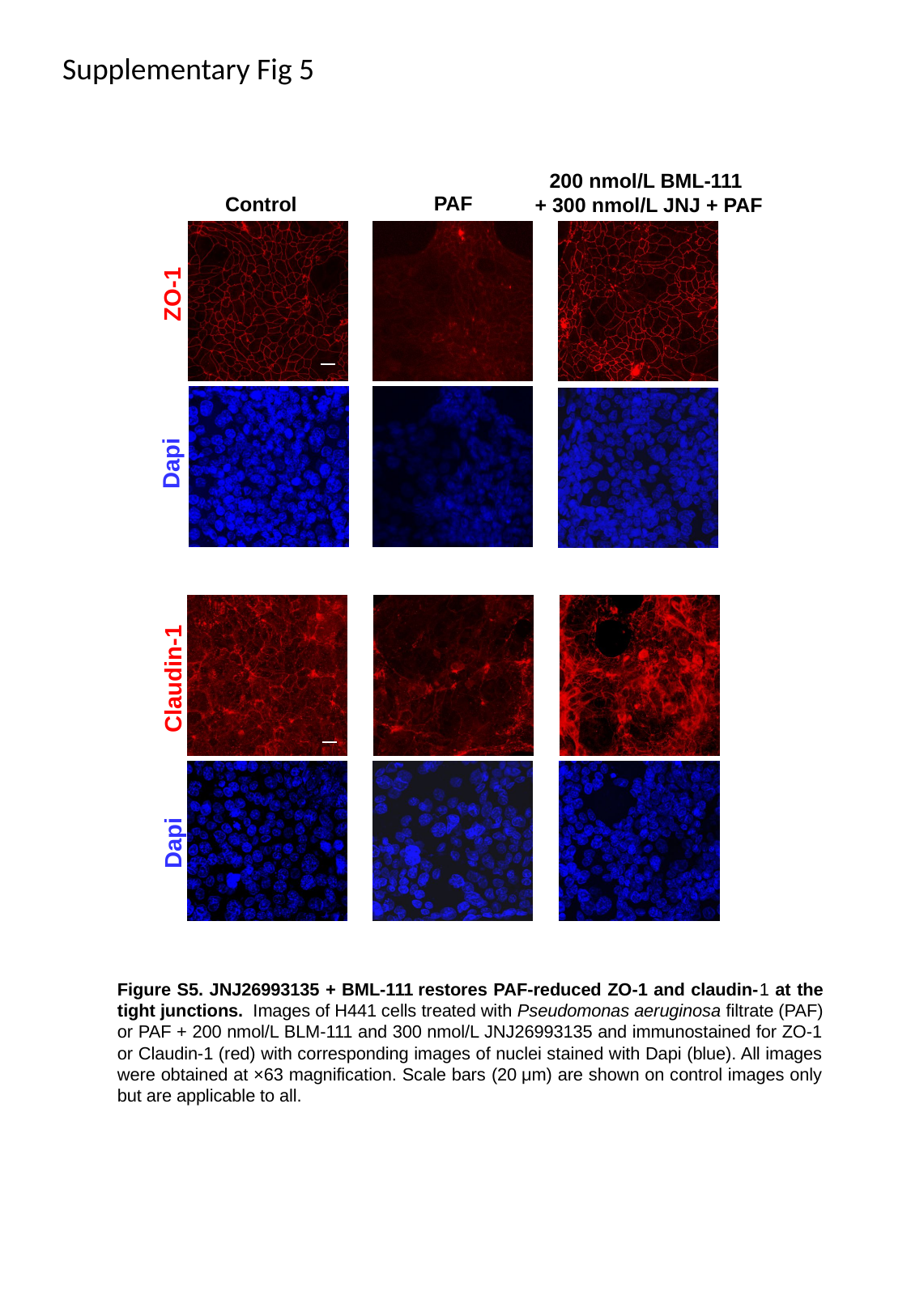

Supplementary Fig 5
200 nmol/L BML-111
+ 300 nmol/L JNJ + PAF
PAF
Control
ZO-1
Dapi
Claudin-1
Dapi
Figure S5. JNJ26993135 + BML-111 restores PAF-reduced ZO-1 and claudin-1 at the tight junctions.  Images of H441 cells treated with Pseudomonas aeruginosa filtrate (PAF) or PAF + 200 nmol/L BLM-111 and 300 nmol/L JNJ26993135 and immunostained for ZO-1 or Claudin-1 (red) with corresponding images of nuclei stained with Dapi (blue). All images were obtained at ×63 magnification. Scale bars (20 μm) are shown on control images only but are applicable to all.
