# Supplementary material for: Lipoxin receptor agonist and inhibition of LTA4 hydrolase prevent tight junction disruption caused by P. aeruginosa filtrate in airway epithelial cells
Source: PLoS One. 2023 Jul 5;18(7):e0287183. doi: 10.1371/journal.pone.0287183 (PMC10321624; doi:10.1371/journal.pone.0287183)
Supplement: S6 Fig — (PPTX) [file pone.0287183.s006.pptx]

## Slide 1
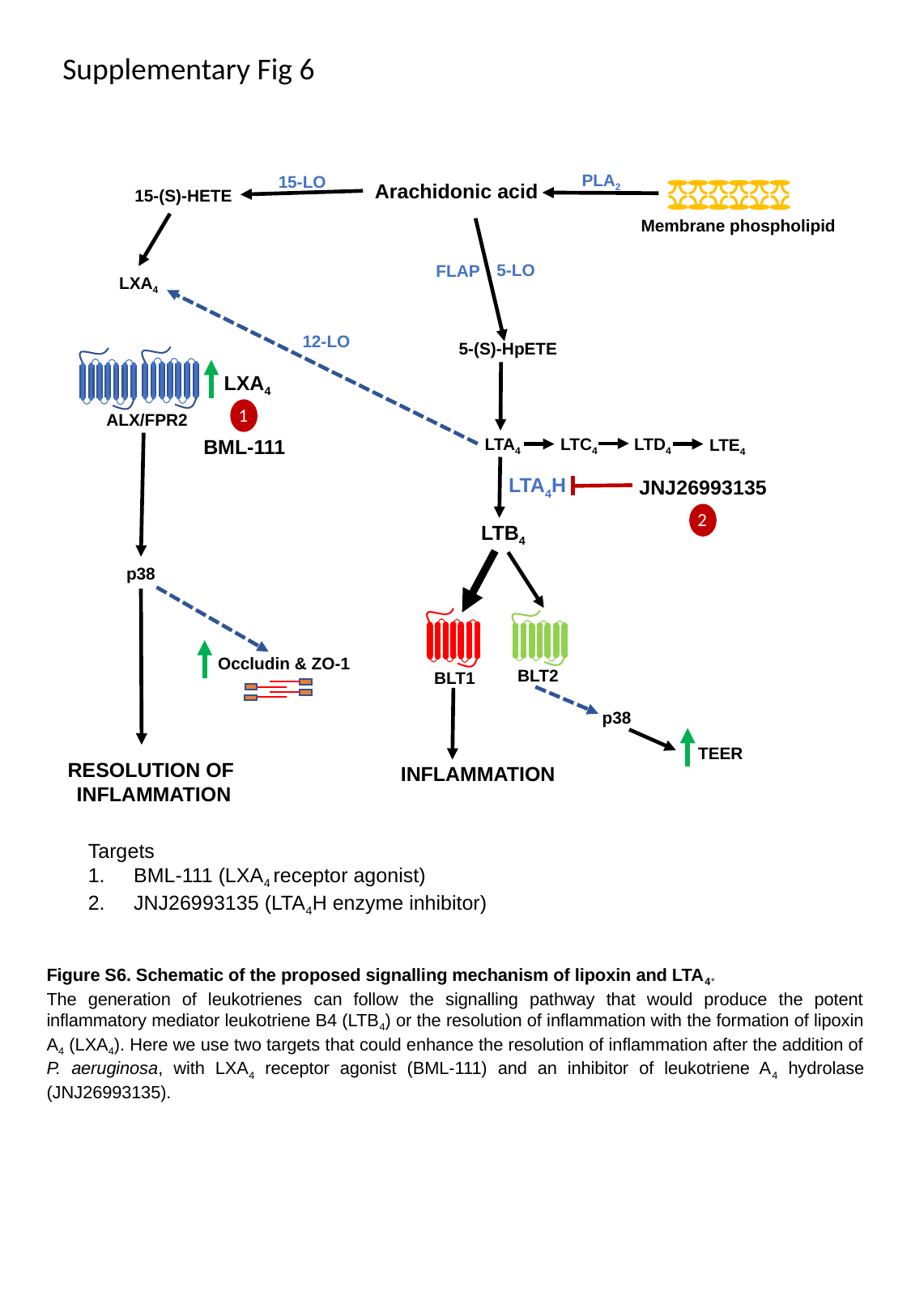

Supplementary Fig 6
PLA2
15-LO
Arachidonic acid
15-(S)-HETE
Membrane phospholipid
5-LO
FLAP
LXA4
12-LO
5-(S)-HpETE
LXA4
1
ALX/FPR2
LTA4
LTD4
LTC4
BML-111
LTE4
LTA4H
JNJ26993135
2
LTB4
p38
Occludin & ZO-1
BLT2
BLT1
p38
TEER
RESOLUTION OF
INFLAMMATION
INFLAMMATION
Targets
BML-111 (LXA4 receptor agonist)
JNJ26993135 (LTA4H enzyme inhibitor)
Figure S6. Schematic of the proposed signalling mechanism of lipoxin and LTA4.
The generation of leukotrienes can follow the signalling pathway that would produce the potent inflammatory mediator leukotriene B4 (LTB4) or the resolution of inflammation with the formation of lipoxin A4 (LXA4). Here we use two targets that could enhance the resolution of inflammation after the addition of P. aeruginosa, with LXA4 receptor agonist (BML-111) and an inhibitor of leukotriene A4 hydrolase (JNJ26993135).
